# Epicutaneo‐caval catheter occlusion in neonates without heparin infusion during parenteral nutrition: A descriptive cohort study

**DOI:** 10.1002/jpen.70037

**Published:** 2025-12-12

**Authors:** Vito D'Andrea, Giorgia Prontera, Cecilia Monachini, Micaela Cerreti, Francesca Baldo, Giovanni Barone, Giovanni Vento

**Affiliations:** ^1^ Neonatology Unit, Department of Woman and Child Health and Public Health Fondazione Policlinico Universitario “Agostino Gemelli” IRCCS Rome Italy; ^2^ Neonatal Intensive Care Unit Infermi Hospital, AUSL Romagna Rimini Italy; ^3^ Neonatology Unit Università Cattolica del Sacro Cuore Rome Italy

**Keywords:** catheter occlusion, central line maintenance, epicutaneo‐caval catheter, heparin, neonates, NICU, parenteral nutrition

## Abstract

**Background:**

Heparin is frequently infused in neonatal central venous catheters to prevent occlusion during parenteral nutrition despite limited evidence of its effectiveness and potential safety concerns in preterm infants. This study evaluated the incidence of catheter occlusion in epicutaneo‐caval catheters managed without heparin in a large neonatal cohort.

**Methods:**

We conducted a descriptive cohort study including all neonates admitted to the neonatal intensive care unit who required epicutaneo‐caval catheters placement for parenteral nutrition. No heparin was added to infusions. Data were collected on catheter characteristics, insertion parameters, and reasons for removal. The primary outcome was the incidence of catheter occlusion per 1000 catheter‐days.

**Results:**

A total of 357 neonates with 357 epicutaneo‐caval catheters were analyzed, representing 4007 catheter‐days. Mean ± SD gestational age was 29 ± 4.1 weeks, and mean ± SD birthweight was 1050 ± 797 g. The mean ± SD catheter dwell time was 11.2 ± 7.7 days. Most catheter insertions occurred in the upper extremities (92.7%), with prematurity being the most common indication (53.7%). Catheter removal was elective in 67.5% of cases. Only three occlusions were reported, corresponding to 0.7% of catheters and 0.76 occlusions per 1000 catheter‐days.

**Conclusion:**

This study demonstrates a low rate of epicutaneo‐caval catheters occlusion in neonates receiving parenteral nutrition without heparin infusion. These findings support the safety and feasibility of a heparin‐free approach in neonatal central catheter management when standardized care protocols are followed.

## INTRODUCTION

Central venous access is essential for the delivery of parenteral nutrition in preterm and critically ill neonates. Epicutaneo‐caval catheters or neonatal peripherally inserted central catheters^1^ are widely used in neonatal intensive care units as they provide reliable long‐term venous access for nutrition and medications. However, because of their small diameter and the administration of hyperosmolar solutions containing lipids and other medications, epicutaneo‐caval catheters are at risk of occlusion, especially with frequent line manipulations.^2^, ^3^ Occlusions remain a major clinical concern, as they can compromise nutrition support, increase the need for additional procedures, and expose fragile patients to further risks. Heparin infusion has traditionally been used to maintain central venous catheter patency in neonates.^4^, ^5^ Yet the evidence supporting its effectiveness in preventing epicutaneo‐caval catheter occlusion is limited and inconsistent.^6^ Some randomized studies suggest a reduction in occlusion rates,^7^, ^8^ whereas others show no clear benefit^9^ and potential harms of heparin such as bleeding or thrombocytopenia^10^ remain a concern. Despite its frequent use, there is a lack of large descriptive studies reporting real‐world incidence of catheter occlusion in neonates receiving parenteral nutrition without heparin infusion. This gap in knowledge hinders the development of evidence‐based recommendations for catheter maintenance in this population. Nevertheless, recent advances in catheter‐insertion techniques, maintenance protocols, and the use of needle‐free connectors may reduce the need for pharmacologic prevention of occlusion.^11^, ^12^, ^13^, ^14^, ^15^ To address this gap, we conducted a descriptive cohort study of neonates requiring epicutaneo‐caval catheters for parenteral nutrition in the neonatal intensive care unit. Our primary aim was to describe the incidence of catheter occlusion in the absence of continuous heparin infusion, under standardized insertion and maintenance protocols. Secondary outcomes included catheter dwell time, total catheter‐days, and reasons for catheter removal. By providing data from a large neonatal cohort, this study contributes new evidence regarding the safety and feasibility of a heparin‐free approach in epicutaneo‐caval catheter management.

## PATIENTS AND METHODS

We performed a descriptive cohort study in 357 neonates who had an epicutaneo‐caval catheter inserted and who received parenteral nutrition at the Gemellli Hospital between January 2020 and January 2025. The inclusion criteria were epicutaneo‐caval catheters with a diameter of 1 French (Fr), administration of parenteral nutrition, and minimum catheter dwell time of 3 days. We included only 1 Fr epicutaneo‐caval catheters because this is the standard device used for neonates in our unit; catheters of larger caliber were excluded to ensure a homogeneous device population. Catheters with a dwell time <3 days were excluded because the aim was to describe outcomes of epicutaneo‐caval catheters intended for sustained parenteral nutrition; catheters removed within 3 days were typically short‐term or technically unsuccessful insertions. The exclusion criteria were catheters larger than 1 Fr, catheters inserted or removed outside the neonatal intensive care unit, and catheters used for purposes other than parenteral nutrition. Continuous heparin infusion has not been part of our routine epicutaneo‐caval catheter maintenance either before or during the study period; therefore, no internal heparinized control cohort is available.

All catheters were managed according to the Safe Insertion of Epicutaneo‐Caval Catheters protocol, a structured insertion and maintenance bundle designed to minimize complications.^11^ The catheters used were 1 Fr polyurethane Vygon Premicath (20 cm length, priming volume 0.09 ml, and flow rate 0.7 ml/min). The infusion line was connected to the catheter via a 4‐cm needle‐free connector with an antireflux valve, and an Alaris CareFusion infusion pump was used for all infusions. The primary outcome was epicutaneo‐caval catheter occlusion, defined as the inability to infuse parenteral nutrition^4^ confirmed by the inability to inject 1 ml of normal saline through a 5‐ml syringe^16^ after standard troubleshooting. The secondary outcomes included catheter dwell time, total catheter‐days, reason for removal, and medications administered in the 48 h preceding catheter occlusion. Elective removal was defined as removal performed when parenteral nutrition was no longer required (full enteral feeds achieved) or when therapy was intentionally completed. Mechanical complications included accidental dislodgement, catheter breakage or leakage, extravasation, persistent kinking, or malposition requiring removal. Standardized maintenance protocols included regular flushing: epicutaneo‐caval catheters were flushed with 0.9% sodium chloride using a standard volume of approximately 0.3 ml delivered with 10‐ml syringes to minimize injection pressure. Prefilled saline syringes were used whenever available to reduce manipulation and contamination risk. All catheters were handled using sterile technique and needle‐free connectors. According to our internal protocol,^17^ all epicutaneo‐caval catheter malfunctions are evaluated by ultrasound to verify catheter tip position before removal. In addition, epicutaneo‐caval catheters are routinely monitored by ultrasound at 48 h and at 7 days after insertion and after any invasive procedures, dressing changes, or manipulations that could affect catheter position.^11^, ^14^, ^17^ This systematic approach aims to promptly detect and correct tip migration, reducing the likelihood of malfunction or related complications.^18^

As per unit protocol, epicutaneo‐caval catheters are removed once neonates achieve full enteral feeding or an oral intake of approximately 100 ml/kg/day (with thresholds adjusted for gestational age). Consequently, infusion rates are never reduced below 0.5 ml/h during catheter dwell.

Descriptive statistics are presented as number (percentage) for categorical variables, mean (SD) for approximately normally distributed continuous variables, and median (IQR) for skewed continuous variables. The primary outcome (incidence of epicutaneo‐caval catheter occlusion) was calculated as the number of occlusions per 1000 catheter‐days. Our study was approved by the Ethics Committee of the Fondazione Policlinico Universitario A. Gemelli IRCCS, Rome, Italy (ID 4880), adhering to the Declaration of Helsinki principles. Written informed consent was obtained from the parents of all patients for the participation and publication of this study.

## RESULTS

A total of 357 neonates were enrolled in this study, and 357 epicutaneo‐caval catheters were analyzed, accounting for 4007 catheter‐days (Table 1). The mean (SD) birthweight of the cohort was 1050 (797) g, and the mean (SD) gestational age was 29 (4.1) weeks. Most catheters were placed in neonates born between 29 and 33 weeks of gestation (34.5%) followed by those born at 26–28 weeks (34.1%). Catheter insertion occurred on median (IQR) at 6 (4–7) days of life, with a mean (SD) weight at insertion of 1010 (800) g. Most epicutaneo‐caval catheters were placed in the upper extremities (92.7%), followed by the lower limbs (4.5%) and scalp veins (2.8%). The main indication for catheterization was prematurity (53.7%), followed by respiratory distress (12.6%) and infection conditions (10%). The mean (SD) catheter dwell time was 11.2 (7.7) days.

**Table 1:** Demographic and catheter characteristics of the study population.

Variable	Value
Patients, *N*	357
Birthweight, mean (SD), g	1050 (997)
Gestational age, mean (SD), years	29 (4.1)
GA ≤ 25 weeks, *n* (%)	52 (14.6)
GA 26–28 weeks, *n* (%)	122 (34.1)
GA 29–33 weeks, *n* (%)	123 (34.5)
GA 34–36 weeks, *n* (%)	29 (8.1)
GA ≥ 37 weeks, *n* (%)	31 (8.7)
Catheters included, *n*	357
Weight at insertion, mean (SD), g	1010 (800)
Day of life at insertion, median (IQR)	6 (4–7)
Catheter indication, *n* (%)	
Prematurity	192 (53.7)
Distress	45 (12.6)
Infection	36 (10)
IUGR/SGA	35 (9.8)
Surgery	22 (6.1)
Asphyxia	19 (5.3)
Other	8 (2.2)
Insertion site, *n* (%)	
Upper extremities	331 (92.7)
Lower extremities	16 (4.5)
Scalp veins	10 (2.8)
Catheter dwell time, mean (SD), days	11.2 (7.7)
Total days of catheter	4007
Reason for removal, *n* (%)	
Elective	241 (67.5)
CLABSI	27 (7.5)
Infection	42 (11.7)
Mechanical complication	44 (12.3)
Occlusion	3 (0.7)
Occlusions per 1000 catheter‐days	0.76

Abbreviations: CLABSI, central line–associated bloodstream infection; GA, gestational age; IUGR/SGA, intrauterine growth restriction/small for gestational age.

Regarding catheter removal, 67.5% were removed electively, whereas 11.7% were removed because of infection, 12.3% because of mechanical complications, 7.5% because of central line–associated bloodstream infection, and only 0.7% because of occlusion. The overall occlusion rate was 0.76 per 1000 catheter‐days. In addition to parenteral nutrition, during the 48 h preceding occlusion, two of three occluded catheters were also used to infuse furosemide, dexmedetomidine, and vancomycin. In one occluded catheter only furosemide, parenteral nutrition, and antibiotic therapy were administered during the 48 h preceding occlusion.

## DISCUSSION

The study aimed to evaluate the incidence of epicutaneo‐caval catheter occlusion in neonates receiving parenteral nutrition without continuous heparin infusion. Our findings showed a low occlusion rate of 0.7%, corresponding to 0.76 occlusions per 1000 catheter‐days, suggesting that heparin‐free management of epicutaneo‐caval catheters can be safe and effective when standard maintenance protocols are applied. These results align with emerging evidence challenging the routine use of heparin in neonatal central venous access. In the heparin infusion for peripherally placed percutaneous central venous catheters study,^7^ heparin infusion significantly reduced catheter occlusion rates (31% in the placebo group vs 6% in the heparin group); however, the baseline occlusion rate in the placebo group was unusually high compared with more recent studies and current clinical settings. Moreover, the study focused on peripherally placed percutaneous central venous catheters, which may differ from epicutaneo‐caval catheters in terms of mechanical and flow characteristics. More recently, a noninferiority randomized controlled trial by Li et al.^19^ employed scanning electron microscopy to assess intraluminal obstruction at the catheter tip in neonates with and without heparin infusion. Their findings demonstrated that parenteral nutrition without heparin was noninferior to parenteral nutrition with added heparin in terms of catheter obstruction and patency duration. Notably, the reported clinical occlusion rates were 4.35% in the heparin group and 11.1% in the no heparin group, although the absolute numbers were low and not statistically significant.

Compared with both studies, our observed occlusion rate of 0.7% is lower despite the absence of heparin infusion. This may be attributed to the use of consistent catheter care protocols, including standardized insertion techniques, the use of needle‐free connectors, and dedicated line‐flushing practices.^11^ It also suggests that mechanical and procedural factors, rather than pharmacological prophylaxis, may play a more critical role in preventing epicutaneo‐caval catheters occlusion in neonates.

Importantly, in two of three occluded catheters, a combination of vancomycin, dexmedetomidine, and furosemide had been administered within the 48 h preceding the occlusion event. These drugs are known to have potential incompatibilities, particularly vancomycin and furosemide, which may precipitate when co‐infused because of pH differences and chemical instability. Dexmedetomidine may further contribute to changes in solubility or flow characteristics, especially in small‐diameter, low‐flow catheters such as epicutaneo‐caval catheters. These observations strongly suggest a possible role of physicochemical drug interactions in the mechanism of catheter occlusion. This combination raises concerns about drug incompatibility, which may have contributed to early intraluminal precipitation and mechanical obstruction. The literature supports this concern. According to pharmaceutical compatibility references and Infusion Nurses Society guidelines,^4^ vancomycin and furosemide are known to be incompatible when administered via Y‐site because of pH‐dependent precipitation risks. Dexmedetomidine, a sedative with slightly acidic pH and potential binding interactions,^20^ further complicates the solution's stability in confined catheter lumens. In neonatal epicutaneo‐caval catheters, which have small diameters and low flow, any physicochemical instability may rapidly result in crystalline deposits or drug precipitates, leading to functional catheter occlusion. Although the number of occlusion cases was limited, and statistical power for categorical variables was low, these findings underscore the need to integrate drug compatibility assessment into routine catheter management protocols. In neonatal care, especially where multiple medications are administered concurrently, this factor may be equally or more important than thrombotic or mechanical causes of occlusion. Although many studies have focused on thrombotic mechanisms of catheter occlusion, these findings highlight that nonthrombotic causes, particularly chemical incompatibility, are equally significant.

As the Li et al. trial showed,^19^ even in the presence of heparin, catheter tips exhibited varying degrees of obstruction on scanning electron microscopy, suggesting that heparin cannot prevent occlusions caused by precipitates or insoluble complexes. Taken together, our findings suggest that line patency is multifactorial, and that the compatibility of co‐infused medications must be carefully evaluated as part of any catheter maintenance strategy. The observed low occlusion rate in our cohort, despite the lack of heparin, emphasizes the effectiveness of standardized catheter‐handling protocols, such as proper flushing, sterile technique, and the use of dedicated lines for incompatible medications.

A limitation of our study is that detailed drug‐infusion data were available only for catheters that developed occlusion, and systematic information was not collected for the remaining catheters. As a result, we were unable to estimate the relative risk of occlusion associated with specific drug combinations. From a methodological perspective, the absence of denominator data prevents calculation of incidence rates or formal statistical comparisons; therefore, these findings should be considered hypothesis‐generating. As a descriptive study our work is not designed to analyze exposure‐outcome relationships. Future studies designed in neonates to compare epicutaneo‐caval catheter occlusion rates with and without heparin infusion during parenteral nutrition are would be ideal. Finally, avoiding heparin use eliminates risks such as heparin‐induced thrombocytopenia^10^ and intracranial hemorrhage. Both complications, although rare, can have severe consequences in preterm neonates and justify a cautious, evidence‐based approach to heparin use.

Given these considerations, our data strongly support a heparin‐free strategy, complemented by rigorous assessment of medication compatibility and strict line‐management protocols. They also reinforce the growing body of evidence suggesting that routine heparin infusion may not be necessary to maintain catheter patency in epicutaneo‐caval catheters used for parenteral nutrition in neonates, provided that best practices in catheter management are followed. However, given the lack of a heparinized control group and the single‐center design, these results should be considered hypothesis‐generating. Further multicenter comparative studies are warranted to confirm these observations.

## AUTHOR CONTRIBUTIONS

Vito D'Andrea contributed to the conceptualization, original draft, and methodology. Giorgia Prontera contributed to the conceptualization, original draft, and methodology. Cecilia Monachini contributed to the investigation and formal analysis. Micaela Cerreti contributed to the investigation and formal analysis. Francesca Baldo contributed to the investigation and formal analysis. Giovanni Barone contributed to the formal analysis and review and editing. Giovanni Vento supervised the study.

## CONFLICT OF INTEREST STATEMENT

None declared.
